# Indigenous plant protection practices of Tripura, India

**DOI:** 10.1186/s13002-021-00476-7

**Published:** 2021-08-13

**Authors:** Satyapriya Singh, Biswajit Das, Anup Das, Sujan Majumder, Hidangmayum Lembisana Devi, Ranjeet Singh Godara, Alok Kumar Sahoo, Manas Ranjan Sahoo

**Affiliations:** 1 ICAR-IIHR-Central Horticultural Experiment Station, Bhubaneswar, 751019 India; 2 ICAR Research Complex for NEH Region, Tripura Centre, Lembucherra, 799210 India; 3 ICAR-Indian Institute of Vegetable Research, Varanasi, 221305 India; 4 ICAR-Indian Agricultural Research Institute, New Delhi, 110012 India; 5 ICAR-IIHR-Central Horticultural Research Institute, Bhubaneswar, Odisha 751019 India

**Keywords:** Eco-friendly approach, Indigenous knowledge system, Insect, Plant protection, Tripura

## Abstract

**Background:**

Traditional plant protection strategies have an integral part of food production system in North Eastern state Tripura, India, which has bestowed with rich heritage and biodiversity. However, there is no comprehensive report on the indigenous plant protection practices (IPPPs) specific to insect and vertebrate pest management, being followed by the inhabitants of the region for centuries. The present study was conducted to investigate, collect, and document the vulnerable IPPP practices followed by the native people from far flung locations of the Tripura.

**Methods:**

The study aimed to document the IPPP following semi-structured questionnaires, participatory interaction, and direct observations with a total of 200 informants. We have calculated the relative frequencies of citation (RFC) for IPPP and estimated principal component analysis to link the status of IPPP with socio-demographic factors of the informants. The relationship between the field of IPPP used and different covariates (age, education, occupation, gender, location, and house type) was assessed using the Kruskal–Wallis test and Chi-square test. The relationship between adoption level and the respondents’ characteristics was analyzed using count regression analysis.

**Results:**

The study found that the status of the IPPP has increased for mitigating pest issues. A total of 39 indigenous practices were recorded specifically to pest management from the ethnic people of Tripura, India. People acquired pretty knowledge about IPPP, and these were inherited from ancestors. The respondents in the study developed notable innovations for the management of many pest issues using locally available resources that warrant cost-effective and eco-friendly. Seed drying before storage to protect grain commodities was the most cited IPPP with a frequency of citation 0.675. In the field of IPPP used, the people primarily practiced agriculture + horticulture + storage category. An important implication from the study is the identification of two IPPP strategies in this region for the first time. Furthermore, the recorded IPPP used field was significantly associated with age, education, occupation, gender, locality, and house type. Likewise, the respondents’ socio-demographic variables were coupled considerably with the adoption of specific IPPP.

**Conclusion:**

The reported IPPP for alleviating pest problems reflects the wisdom and generosity of the ethnic growers of Tripura, India. The study suggests the IPPP has strong potential in an integrated pest management approach passed down from generation to generation. The vulnerable practices largely remained unexplored due to inadequate scientific scrutiny and authenticity, yet in danger of being lost if not documented systematically. This study provides the first step toward accessing the valuable technology of untapped Tripura in IPPP and could be viable in paving action paradigm for their preservation, diffusion, and application with advanced pest management options.

## Introduction

Indigenous plant protection practice (IPPP) assembles awareness and understanding of various facts related to pest management that farmers have developed over a long period and continue to expand and diffuse across communities. Since the inception of agricultural practices, there has been a constant struggle between mankind and pests for better crop yield and survival. Dependency on synthetic chemical pesticides by growers has an adverse impact on health and the ecological balance [1]. Though chemical pesticides are giving immediate pest control, the traditional knowledge stands well in its position when ecology and sustainability considered as a whole. In this perspective, north-east Himalayan region (NEH) is the mega preserver of these indigenous knowledge systems [2]. Being considered the biodiversity hub (within the Indo-Burma region) of the world, the north-eastern region of India, particularly Tripura, is bestowed with rich natural resources and conserves various age-old practices [3].

Tripura is a state located in the north-eastern part of India, covering an area of 10,492 km^2^, of which 60 percent constitute forest cover and the remaining 40 percent is available for cultivation. It is situated at 22° 56′ to 24° 32′ latitude and 91° 10′ to 91° 10′ to 92′ 21′ longitude. It has strategic importance because it shares international boundaries with Bangladesh mostly 80 percent of its periphery and states such as Mizoram and Assam. It enjoys a humid-mild tropical climate with average annual rainfall 2400–2500 mm, RH 70–85%, temperature 10 °C–35 °C, and pre-monsoon storms in March-June followed by heavy monsoon rain during July–September–October. More than seventy percentages of the people of Tripura practice agriculture as their sole source of livelihood, where small and marginal farmers contribute about 95 percent of the total farming community. Although 40 percent is the cultivable land of the total area, still the economy of the entire strata is majorly agrarian. Agriculture is an important sector, which accounts 26 percent of the state gross domestic products (GDP). However, off late due to immense pressure on land as the state has a very high population density limited the average landholding size, i.e., only 0.97 ha, which is the lowest among the seven other north-eastern Indian states.

In general, the land of this region is inaccessible, marginal, and less crop productive compared to the main land [4]. Besides general agricultural practice in plain land, the ethnic people majorly adopted two farming systems, viz*. jhum* or shifting cultivation and terrace or wet cultivation. Shifting cultivation or slash-and-burn agriculture (commonly known as *jhum*) is a major farming system in which farmers rotate land rather than crops to sustain livelihood [5], similar to strategies used in Africa, some parts of Europe and southeast Asia [6]. The area under such lands is cleared once in five to eight years for better crop production. It is realized that *jhum* practice, a traditional and dominant cultivation method, has been balanced with the environment for centuries in Tripura. In the terrace method, the entire hill surface is cut into many terraces, irrigation by a network of water channels that flow down from one terrace to other. It is an easier method of cultivation as compared to *jhum.* However, due to wide altitudinal variations, terrace cultivation is found in some rural pockets. Rice is the major crop and staple food of the Tripura people, although certain cereals and many vegetables are also cultivated. As hill agriculture is a dominant enterprise in the region which is comparatively more prone to insect pest infestation due to pleasant climatic conditions, it poses serious problems in protecting the crop and achieving good productivity in diverse crops. The cultivation methods are mostly eco-friendly and tuned to the need of the local people. Utilization of plants and animal parts and products is the valuable component of indigenous knowledge in the management of pests and diseases of crops, particularly in *jhum* system [7]. The uniqueness of this knowledge is that it is ecologically affordable, socially acceptable, economically viable, and environmentally sustainable [8, 9]

The treasurer land of Tripura conserves various pesticidal plants and a handful of technologies for pest management through indigenous means. Through trial and error, farmers have developed many management practices traditionally to protect crops from various pests and diseases. Transcription and transmission of such age-old knowledge from generation to generation are most commonly found in this area with the undeveloped backgrounds. Nonetheless, scientific validation, systemic incorporation, and sustainable application of IPPP on insect pest management in light of modern technology are the challenges of the present scientific era. To meet these challenges, a strong and information-rich data bank is required to be built. Concerning natural resource-richness and existing indigenous knowledge, the Tripura region of northeastern part of India has a clear-cut competitive advantage globally. Crop diversity *vis-a-vis* insect pest diversity exhibits higher magnitude in Tripura. Occurrence, population density, varying life cycle stages, and host specificity of various pests present a mammoth challenge to the scientific community for formulating environmentally sustainable management methods. Transfer of technology from the laboratory to the farmers’ field also requires enough time and opportunity. This backdrop presented the platform for IPPP to take over the stage as the easiest and earliest available remedy to tackle the insect pest problems. Undoubtedly, the benefits of technology should be handed over to the rural poor across the country and the diffusion of sustainable technology among needy growers. The World Summit on Sustainable Development held at Johannesburg in South Africa in 2002 has strongly advocated using the local indigenous knowledge in crop husbandry practices.

Although ethnic groups reported a scattered knowledge of the indigenous insect pest management practices from northeastern India [7, 10], detailed synthesized information of the Tripura region remained unexplored and largely obscured. Thus, there is a scope for using them to develop many lowcost eco-friendly pestmanagement strategies. Although the advanced technology of pest management has been globally disseminated, tailoring the knowledge, including coordinating with indigenous knowledge for practical application at the local level, is scare and getting momentum slowly but steadily among the end-users. Hence, in this backdrop, the current study aimed to investigate carefully, collect, analyze, and record the indigenous knowledge about plant protection practices followed by ethnic growers of Tripura that may combat the insect pests’ issues in various agro-ecosystems. Therefore, the present study was undertaken to the systematically synthesize IPPP practiced in this region. This approach could be the first report of the IPPP from Tripura. The present study focused on the hypothesis that the IPPP used field and adoption level of respondents as a function of informant-specific explanatory variables such as age, education level, occupation category, gender, location, and house types. Moreover, this synthesized collection of protection measures followed by local people will help to propose an action paradigm for preservation, diffusion, and extension of desirable insect pest management tactics for the benefit of the local community, farmers of the nation, and the globe. These sustainable technologies could provide the key guidance for an effective and sustainable solution for agricultural insect pest management. Further, it could be incorporated to advance pest management practices to enhance its efficiency for assured and eco-friendly food production.

## Research methodology

### Study site and data collection

In order to understand the IPPP followed by the ethnic groups of Tripura, the present study was conducted from April 2019 to July 2020 involving 40 locations across eight districts of Tripura, viz. Sipahijala, Khowai, Gomati, North Tripura, South Tripura, Unnokoti, West Tripura, and Gomati. The target groups for the study were both hilly and plain areas that signify practical application in both upland and low-land ecosystems in other places. The survey was conducted after getting ethical approval through verbal consent from informants. The data about IPPP and related local knowledge were collected using field surveys, open interviews, and semi-structured questionnaires. All the activities were carried out with informed consent. Farmers were selected randomly in each location, and active participation was ensured through open interactions.

Furthermore, field visits also encountered to find out the existence of the technology. A total of 200 informants (81 females and 119 males) aged between 21 and 80 were interviewed. The informants were categorized into four groups such as non-educated (24.5%) and education of primary level, i.e., up to class five (27%), secondary level, i.e., class six to ten (34%), and graduates (14.5%). Further, the majority (90%) of the respondents interviewed were marginal farmers, i.e., less than one hectare land. It is also noticed that 84.50% of the informants were dependent on agriculture as their sole profession, whereas 15.50% were having allied activities like shops, small businesses, and services in both government and private sectors. The detailed demographic properties, including educational level, occupation, gender, age group, location of the study area, and house type, are presented in Table 1. Furthermore, the information on plant protection strategies, local name, location/crop, procedure/method of application, target pest, and rationale is recorded (Table 2). The semi-structured interviews were performed based on the method described by Deka et al. [7] with minor modifications. Moreover, prior to the survey, the questionnaire was pre-tested with farmers in Lembucherra, Tripura, extension experts, and plant protection specialists and necessary refinement was made.

**Table 1:** Demographic profile of informants (n = 200)

Characteristics	Description	Number	Frequency (%)
Age	21–30	23	11.5
	31–40	31	15.5
	41–50	39	19.5
	51–60	46	23
	Above 60	61	30.5
Education	Not educated	49	24.5
	Primary	54	27
	Secondary	68	34
	University	29	1405
Occupation	Agriculture	169	84.5
	Non-agriculture (business, job, shop etc.)	31	15.5
Gender	Male	119	59.5
	Female	81	40.5
Location	Hill	142	71
	Plain	58	29
House type	Kuccha	134	67
	Pucca	66	33

Kuccha: A kind of house, where the walls are made up of bamboo, mud, grass, stones, thatch, straw, and unburnt bricks; Pucca: Dwelling place considered to be solid, made up of stone, brick, cement, concrete, etc.

**Table 2:** IPPP in Tripura

Sl. No	Technology/plant protection methods	Crops/ location	Procedure/use	Target pests	Rationale	FC	RFC
A	**Usage of home-utilized products or waste materials and appliances**						
1	Ash application [Vernacular language: (Bangla—Chai/Chali, Kokborok—Thapla)]	-Amaranthus, vegetable crops, kitchen garden -Legume crop storage	-Application of wood ashes on crops and around the crop - Ashes mixed with pulse seeds	-Chewing pests like beetles (e.g., blister beetle *Mylabris phalerata*), termites (*Odonotermes spp.*) -Bruchids	Abrasion of mouth parts, particularly mandibles, repels insects due to irritation [40] -Physical barrier in oviposition [41]	109	0.545
2	Tin boxes like oil cane, amul cane or any food supplement box (Fig. 4)	Coconut	Banding the cut open tin on stem near ground region	Rats: coconut rat: *Bandicota indica* (Burrowing rat) Squirrels: (*Funambulus spp.)*	Slippery surface of the substrate reduce the frictional force resulting barrier for climbing [64]	44	0.22
3	Tying ribbons, cassette reels or plastic bags (Fig. 3a)	Maize, rice	Colored ribbon or cassette reels or plastic bags, particularly during grain filling stage	Grain feeding bird, viz. common pigeon or blue rock pigeon (*Columba livia*)	Acts as bird scarrer [65]	19	0.095
4	Placing fish nets	Nursery seed beds or main field	Immediately after sowing the fish nets are covered over it and fixing with bamboo pegs	Birds like sparrow (*Passer domesticus*), pigeon (*Columba livia*)	Physical barrier check the lifting and feeding of sowing seeds [66]	25	0.125
5	Dhup/Dhoya (coconut husk or paddy husk or maize cobs)	Citrus orchard	Smokes produced by burning coconut husk or paddy husk	Fruit sucking moth (*Eudocima fullonia*)	Acts as repellent [67]	38	0.19
6	Soap and detergent	Vegetables	Foams of the products applied on plant	Sucking pests, viz. whitefly ( *Bemisia tabaci* Gennadius), leaf hopper (*Amrasca biguttula biguttula* Ishida) etc.	Creates slippery surface acts as non-host plant, i.e., antixenosis [48]	6	0.03
7	Hookah water (Fig. e,f)	Kitchen garden vegetable	After consumption, the left-out water is used	Pod borers (Bhendi pod borer, *Earias vitella* Fab., sucking bugs (*Riptorus spp., Clavigralla spp.*) of vegetables etc	Repellent mechanism [37]	98	0.49
B	**Food Products**						
8	Shaft of jackfruit	Cucurbits, fruit crops	Placing the middle portion of jackfruit (shaft) in gourd types crops and also in mango, guava orchard	Fruit fly (*Bactrocera spp.*)	Sweet smell lure and attract the pest for oviposition and the sticky surface glued with ovipositor of fruit fly leads to killing of insects (act as trap)	16	0.08
9	Placement of pomelo/citrus	Rice	Cut open piece of citrus/pomelo placed in rice field	Stem borer, *Scirpophaga incertulas* (Walker)	Repellent action [51]	11	0.055
10	Jackfruit latex	Paddy field	Putting bamboo sticks with jackfruit latex around the rice field	Rat (*Bandicota indica*)	Rat used to stuck in sticky late [50]	9	0.045
C	**Use of organisms/birds**						
11	Erection of bird perches	Brinjal, rice, maize, cruciferous crops, sorgum, pearl millet	Putting bamboo stem on field having many lateral branches	Brinjal fruit and shoot borer (*Leucinodes orbonalis* Guenee), rice stem borer, *Scirpophaga incertulas* (Walker) (particularly vegetative stage), different lepidopterous larvae infesting in cruciferous crops, stem borer in millets and maize (*Chilo spp*.)	Attract carnivorous birds for rest and promote prey searching; Larvicidal properties and hydrocyanic acid content of bamboo boost the pest management strategy [68]	75	0.375
12	Duck rearing	Rice	Duck rearing near rice field encourage predation	Rice hispa, *Dicladispa armigera* (Olivier)	Biological control agent [69]	34	0.17
D	**Animal products and wastages**						
13	Cow dung slurry	Moringa	Putting the cow dung slurry on top surface of the rejuvenated moringa tree	Borer and subsequently check pathogen attack	Creates barrier and check secondary infection [53]	66	0.33
14	Putting rotten crabs or snails or dead frogs	Rice	Placement of crushed and fermented crabs or snails or dead frogs in rice field, particularly during milking stage	Gundhi bug (*Leptocorisa spp.*)	Acts as repellent, foul smell deter the insects [54]	17	0.085
15	Cow dung with water or cow urine	Rice	Suspended slurry application in open field during nursery stage	Thrips, *Stenchaetothrips biformis* (Bagnall)	Odor of cow dung and cow urine acts as repellent [70]	21	0.105
16	Chicken egg shell	Rice, sugarcane and maize	Placement of crushed eggshell as powder	Snails, molluscs, lice, beetles, rats, and ants	Eggshell powder (contain calcium carbonate) acts as repellant [71]	22	0.11
E	**Synthetic products**						
17	Petrol/ kerosene application	Mango, citrus	Injecting kerosene or petrol in stem bore hole and plastered with cement or soil slurry	Mango stem borer; *Batocera rufomaculata* DeGeer, citrus trunk borer: *Anoplophora spp.*	Burning sensation, respiration choking, and killing of insect due to asphyxiation (deficiency in oxygen) [56]	58	0.29
18	Tire burning	Rice	Synthetic tire burning near rice field just before panicle initiation or milking stage	Gundhi bug (*Leptocorisa spp.*)	Strong smell repels the pests [72]	17	0.085
F	**Natural resources**						
i	**Pesticidal plant**						
19	Tobacco leaf (*Nicotiana tabacum* L.) extract application	Brinjal, okra, maize and pulses	Direct application of water extracts of tobacco leaves after boiling	Chewing insects (blister beetle: *Mylabris phalerata*, hadda beetle: *Henosepilachna vigintioctopunctata* (Fabricius)), sucking pests (aphid: *Aphis gossypi* Glover*, Rhopalosiphum maidisi* (Fitch), leaf hopper: *Amrasca biguttula biguttula* Ishida, thrips: *Thrips tabaci* Lindeman, and borer, viz. brinjal fruit and shoot borer: *Leucinodes orbonalis* Guenee, early shoot borer *Earias vitella* Fab	Secondary plant metabolite, i.e., nicotine acts as insecticide [37]	76	0.38
20	Neem leaf (*Azadirachta indica* A. Juss.) extract application	Cereals, pulses, vegetables (almost all crops)	Steam decoction of neem leaves and the water extracts is sprinkled on plants after filteration with normal white cloths	All types of insects (chewing, sucking, borer, miners etc.)	Broad spectrum insecticidal activities having insect growth disruption; oviposition suppressant and sterilant and antifeedant action [37]	108	0.54
21	Neem leaf (*A. indica* A. Juss.)	Stored products	Dried neem leaf placed with stored commodities	Stored grain pest, viz. rice weevil: *Sitophilus oryzae* L., pulse beetle: *Callosobruchus spp.*, Angoumois grain moth: *Sitotroga cerealella* (Oliver), rice moth: *Corcyra cephalonica* (Stainton)	Antifeedant and repellent action keeps the pest away [37]	97	0.485
22	*Holarrhena pubescens* (Buch-Ham) Wall. (Apocynaceae), [Vernacular language: (Bangla- Kurcha gachha/kuchima gachha, Kokborok- kuchimavompang)] (Fig. 2b)	Rice	Placement of twigs in rice field, particularly during vegetative stage	Leaf folder *Cnaphalocrocis medinalis* (Guenee), stem borer: *Scirpophaga incertulas* (Walker)	Conessine, the steroidal alkaloid has repellant action [46]	88	0.44
23	Placing of turmeric leaf (*Curcuma longa* L.) (Fig. 2c)	Rice	Keeping leaf in submerged rice field during vegetative stage	Leaf folder: *Cnaphalocrocis medinalis* (Guenee)	Active principles, viz. alkaloids, tannins, phenolics, terpenoids present in turmeric leaf has repellent action [73]	69	0.345
24	Dried chilli: *Capsicum annum* L., calotropis leaf: *Calotropis gigantea* (L.), tobacco leaf, curry leaf: *Murraya koenigii* Spreng	Stored commodities	Placing with stored grain products during packaging time for long term storage	Stored grain pests	-Chilli, containing capsaicin component having insecticidal activity [35] -calotropis, having active toxic principle, viz. alkaloids repels the insects [36] -tobacco, nicotine as insecticide [37] -curry leaves, repellant and antifeedant action [38]	119	0.595
25	Use of fresh branch of crofton weed, *Eupatorium adhenophorusm* [Vernacular language: (Bangla- pisach)]	Paddy	Branch is cut about 1 m height and placed in rice field @100/ha	Brown plant hopper [*Nilaparvata lugens* (Stal)]	alkaloid compounds, terpenoids etc. [74]	61	0.305
ii	**Miscellaneous**						
26	Bamboo beating or empty drum beating	Brinjal, chilli, maize rice, til, water melon, pumpkin particularly in jhum cultivation	Beating of bamboo sticks nearby field	Grain feeding birds like parrot: *Psittacula spp.*, pigeon: *Columba livia*	Noisy sound threatens the bird acts as bird scarrer [75]	45	0.225
27	Dried colocasia leaves: *Colocasia esculenta* L., grease/castor oil or any sticky substance	Vegetable crops	Yellow dried leaves of colocasia smeared with any sticky material placed in field	White fly: *Bemisia tabaci* Gennadius, aphid: *Aphis gossypi* Glover	Yellow color attracts the sucking pests [58] and the glued to the sticky surface and killed	24	0.12
28	Use of catapault [Vernacular language: (Bangla- guloil/gulti/gulari)]	Fruit crops or cereals crops during grain ripening stage	Throwing stones or any hard material by using catapault	Birds or animals	Scare the target organism [76]	29	0.145
29	Soil application	Maize crop	Dried soil or slurry directly applied on whorl region of maize	Fall armyworm: *spodoptera frugiperda* (J.E. Smith)	Unavailability of free space for movement, lack of oxygen leads to death by asphyxiation	6	0.03
30	Drying of seeds	Grains used for seed and consumption purpose	Post-harvest drying of grains by exposing to sunlight	Stored grain pests	Proper drying helps in cure of field infested commodities by killing hidden stages of insect life cycle and also reduce the moisture level to optimum level which prevents the infestation due to unfavorable state to pest [34]	135	0.675
31	Weed removal	Rice	Removal grassy weeds from rice bunds, viz. *Imperita cylindrica*	Brown plant hopper: *Nilparvata lugens* (Stal)	Destruction of alternate hosts abolish the microhabitat of insects during off season [77]	41	0.205
32	Rotation of crops	Solanaceae/cruciferaceae-pulses-cereals	Avoid monocropping and follow crop rotation procedures like pulses after rice and vegetables after pulses	Monophagous (brinjal fruit and shoot borer: *Leucinodes orbonalis* Guenee, rice stem borer: *Scirpophaga incertulas* (Walker) and oligophagous pest (pulse pod borer: *Maruca vitrata* Fab., Cruciferous head borer: *Hellula undalis* (Fabricius), diamond back moth: *Plutella xylostella* (Linnaeus) etc.	Avoiding continuous supply of food subsequently break the life cycle of particular insect [78]	72	0.36
33	Bird scarer [Vernacular language:(Bangla: Kaktarua)]	Rice, maize, chilli, etc.	Placement of a statue like human, i.e., human effigies (with stick, straw and clothes) in middle of the field or pseudo-human/ghost structure of different kinds using earthen painted pots are used	Grain feeder Birds	Structure threatens the birds as resemblance with human [55]	69	0.345
34	Ploughing	Potato	Deep ploughing with country plough	Potato tuber moth: *Phthorimaea operculella* (Zeller)	Expose pupae to sunlight, dried and killed [79]	13	0.065
35	Sand over potato	Potato	Sand cover of one to two inches over potato in storage condition	Potato tuber moth: *Phthorimaea operculella* (Zeller)	Prevents oviposition [80]	21	0.105
G	**Mixed application**						
36	*Lantana camara* and cow dung	Vegetable cropping system	Stem decoction of *L. camara* mixed with cow dung is applied on plants	Aphid: *A. gossypi* Glover, leaf hopper: *A. biguttula biguttula* Ishida, thrips: *T. tabaci* Lindeman	Bioactive principle of secondary metabolites repels insects [80] (synergized by cow dung) [53]	27	0.135
37	Rope pulling and kerosinized water	Rice	In water logged rice kerosene is mixed with water and rope is pulled across the field	Rice case worm: *Nymphula depunctalis* (Guenee)	Dislodging the pupae through rope pulling in kerosinized water killed insects	19	0.095
H	**Local traps**						
38	Traditional bamboo trap with bait (Fig. 5)	Rice	Bait is placed inside the trap (made in bamboo or woods, dimension: length, 18 inches × width 4 inches)	Rodents	Luring and killing (as rats unable to move backward) [45]	98	0.49
I	**Storage structures**						
39	Granary: Storage structure (Fig. **3****d,g)**	Rice	Bamboo plastered with cow dung or mud slurry using in storage purpose (man height structure in varying dimension) and on the top of which branches with leaves of *Zanthoxylum acanthopodium or Calotropis spp* were placed	Rice weevil: *Sitophilus oryzae* L., rice grain moth:* Sitotroga cerealella* (Oliver)	Well-plastered bamboo prevents entry of insect pests and maintain proper aeration, and the leaves having repellant activities [81]	107	0.535

### Information document and IPPP

We used a portable notebook to record all the information during the interview and then organized them into an Excel sheet (Microsoft Corporation, http://www.microsoft.com) in a synthesized format. The emphasis was given to record all the information provided by the informants in an Excel sheet. Because diverse language prevailed among ethnic groups, the information on IPPP was collected through informal interactions and participatory manner by engaging the local moderator with knowledge in vernacular language. Direct observation was used to record the image of the prevailing technologies in the field with a digital camera, i.e., with the permissions of the informants. Further, hand architect was drawn to represent some strategies informed by the respondents. All the photographs of specific practices referred in this paper and drawn architect was deposited at the Division of Crop Protection, ICAR Research Complex for NEH Region, Tripura Centre and Meghalaya, India.

### Data analysis

Based on questionnaire, preliminary data collected from different locations were cross checked for each IPPP to avoid any discrepancies. Further, all data obtained were compiled, transcribed, and categorized into different streams. Data were analyzed using descriptive and quantitative statistical methods. The status of the IPPPs used for pest management (increase, decrease, same, and never used) was calculated and shown as PCA using XLSTAT Premium 2020.2.1, Adinsoft, NY.

For all the collected IPPPs, frequency of citation (FC), and relative frequency of citation (RFC) were calculated following the reports of Tardío and Pardo-de-Santayana [11].

$${\rm RFC} = {\rm FC/N}$$

FC: number of informants who mentioned use of the particular IPPP strategy and N: total number of informants took part in survey.

Further, the results of the RFC and the best ten IPPPs are presented in the radar diagram using Microsoft Excel, 2010.

The disaggregated information was subjected to the Shapiro test, Kruskal–Wallis test, and Chi-square test for the dependent variable, i.e., field of IPPP used and analyzed using [12]. The Shapiro test was performed to test the normality of the data. The Kruskal–Wallis test was performed to test the relationship between IPPP used fields with age class and occupation levels. The relationship between gender, house type, occupation, and location with the IPPPs used field was statistically analyzed using the chi-square test.

The relationship between the socio-demographic variables and the IPPP adoption was analyzed using count regression analysis. The attempt was made to test the hypothesized relationship of predictor variables such as age, education, gender, location, occupation, and house type with the predicted variable, i.e., number of IPPP adopted by the individual subject. As all counts are positive integers and in rare events, the poisson count regression has been investigated [13].

## Results

**Fig. 1:**
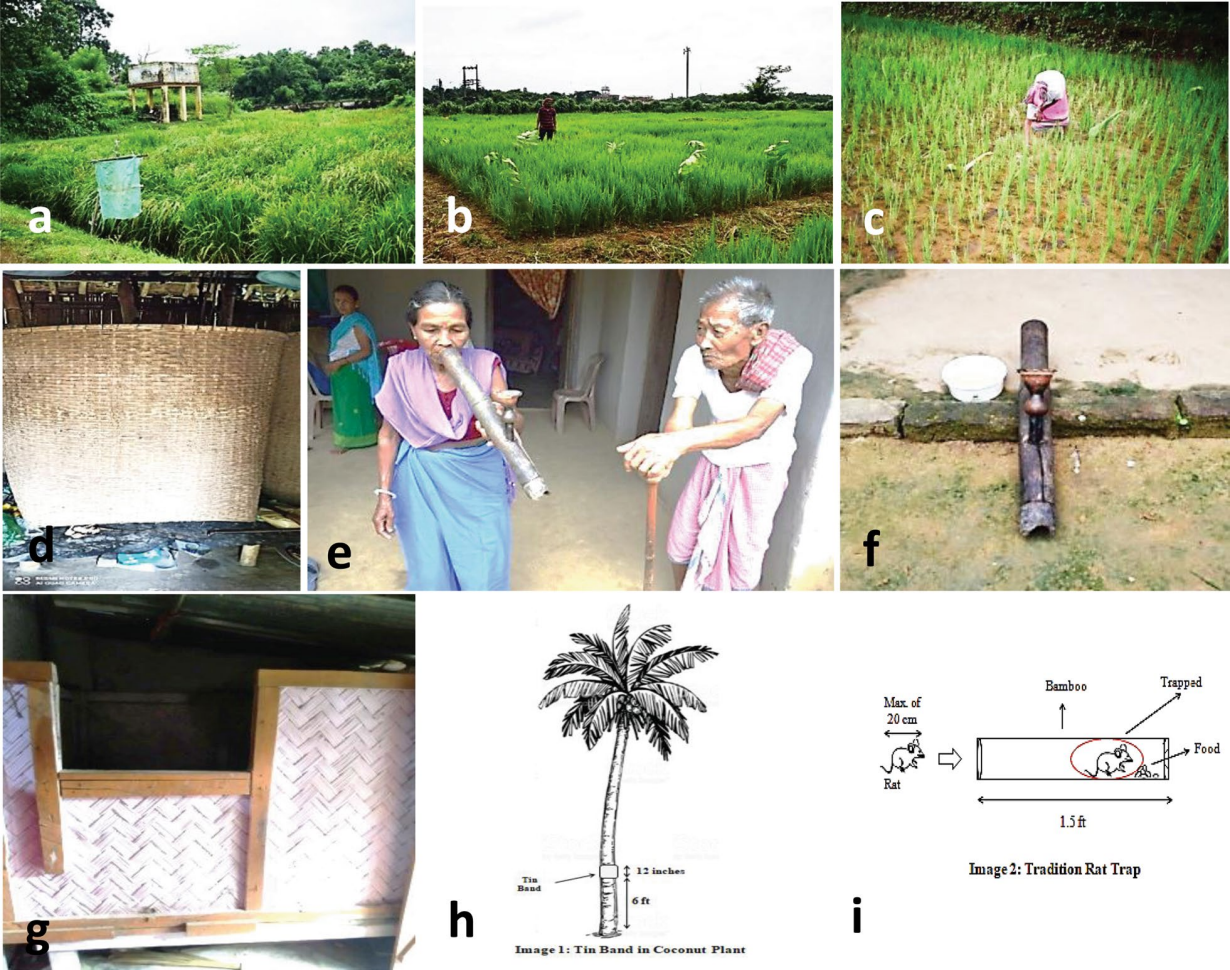
Certain IPPP recorded during field visit.** a** Bird scarrer [Bengali language: Batpataka],** b** Placement of* Holarrhena pubescens* (Buch-Ham) twigs [Bengali:Kurcha gachha/kuchima gachha; Kokborok (an ethnic tribe language): Kuchimavompang] in rice field for the management of Leaf folder* Cnaphalocrocis medinalis* (Guenee), stem borer:* Scirpophaga incertulas* (Walker),** c** Keeping turmeric leaf;* Curcuma longa* L. [Bengali:Haludpata; Kokborok:Sutwi] in rice field to control Leaf folder:* Cnaphalocrocis medinalis* (Guenee),** d** Granary structure for cereal storage [Bengali: Dol; Kokborok: Dol/ Kaniya/Mayam],** e**–**f** Hookah water (consumed tobacco leaf water;* Nicotiana spp*) [Bengali: Hookah; Kokborok: Daba] used against Pod borers (Bhendi pod borer,* Earias vitella* Fab., sucking bugs (*Riptorus spp*.,* Clavigralla spp*.) of vegetables crops,** g** Granary structure [Bengali:Gola; Kokborok: Chapmakampa/Bera] for long term storage of cereal and pulses commodities,** h** Banding of tin on ground region of coconut to prevent climbing of rodents (hand architect),** i** Trapping by luring (rodent trap made up of bamboo, lure: any grain commodities) (hand architect)

In the present age of technology, non-judicious usage of various chemical pesticides and other synthetic materials has casting harmful effects on the environment, leading to hazards of various types like ecosystem disturbance and negative impact on human welfare [1]. As a demand, scientific investigation, documentation, and analysis of IPPP are now being realized and encouraged. The summary sheet (Table 2; Fig. 1a–i) depicts the ethnic groups of Tripura that adopted various indigenous methods to manage insect pests.

### Status of IPPP used

**Fig. 2:**
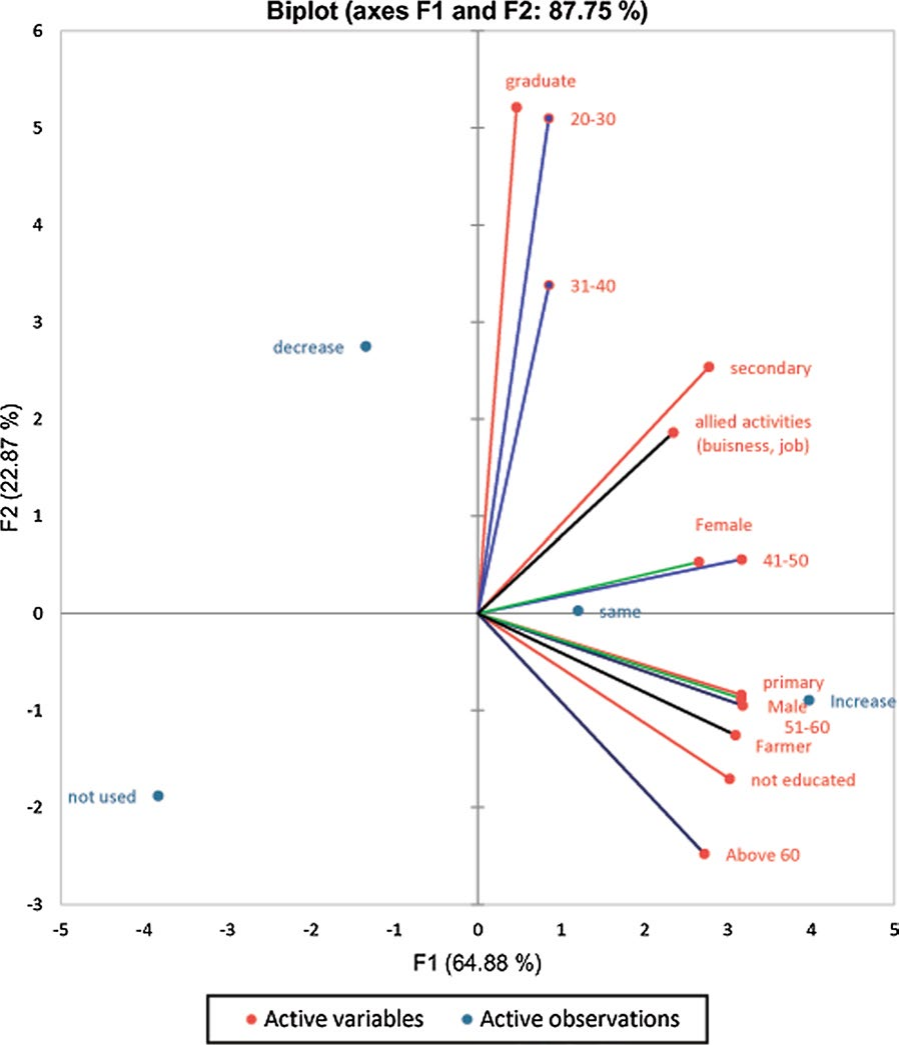
Principal component analysis (PCA) showing the relationship between status of IPPP (i.e., increase, decrease, same, or not used) and respondents’ covariates

The PCA based on the responses of the informants of various explanatory variables with IPPP is demonstrated in Fig. 2. Our analysis revealed that the covariates of various categories respond variably to different levels, i.e., increase, decrease, same, not used for IPPP status to manage pest issues. Among age group (above 60 and 51–60 group), occupation (farmer), education (not educated and primary level), and gender (male) are found to be increase response for IPPP status during present time. For the age group (31–40 and 20–30), the education level (graduate) has given the decrease in response status of IPPP during the current era (Fig. 2).

### Farmers' knowledge of crop pests:

Farmers shared the name of pests that damage their crops. Most of the farmers gave the name of insect pests in the local language. Nearly all farmers mentioned a few insect pest descriptions regarding its damage and losses. Among the insects, stem borer; *Scirpophaga incertulas* (Walker), leaf folder; *Cnaphalocrocis medinalis* (Guenee), and gundhi bug; *Leptocorisa spp.* in rice, fruit, and shoot borer *Leucinodes orbonalis* Guenee in brinjal, and fruit fly; and *Bactrocera spp.* in cucurbits and fruits were commonly mentioned by the farmers. Some maize growers have recently revealed that a pest is attacking their crop, causing defoliation and severe yield losses. It has been identified as fall armyworm (*Spodoptera frugiperda*): an invasive pest. Among non-insect pests, informants noticed birds are the major problem, particularly during grain ripening stages and rodents in stored commodities. The informants also mentioned other factors which damage their crops, viz*.* diseases, water logging, drought, weeds, and wild animals despite insect attack. Among all the stresses experienced by the farmers, the study focused only on insect pests problems.

The detailed investigation found that for the protection of agriculture crops against insect pests in field as well as storage, ethnic people practiced traditional management strategies using readily available resources. The set questionnaire gave clear-cut information regarding IPPP. Participatory interaction with respondents revealed that the IPPP practices are known to them from their ancestors and fellow farmers. It was also observed that most of the IPPP were used in rice crops and in storage protection. In addition, the practices were most followed at kitchen garden pest management. The result was encouraging, where the daily consumption prefers healthy organic products. These indigenous technologies managed both sucking and chewing pests. Variability in doses was not uncommon to a method of application. The remark on each agricultural pest management practice drawn from the informant’s experiences is presented (Table 2). During this study, our major focus was on scientific documentation of evidence of using IPPP in insect pest management from various study places in far-flung localities of Tripura. As a part of the program, photographic documentation was performed, which formed a strong baseline of our data. A glimpse of that effort is presented in Fig. 1a–i. In light of the collected data and survey sheets, facts were scrutinized scientifically, and an environmentally sustainable insect pest management strategy is envisaged as promised to the scientific world.

### The IPPP recorded and relative citation of frequency

**Fig. 3:**
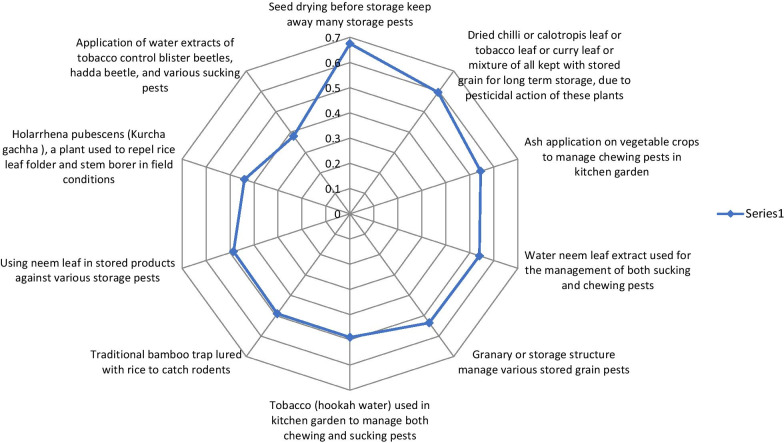
List of top ten ranked IPPP recorded by respondents shown the relative frequency of citation

A total of 39 strategies of IPPP were documented as being perceived by the informants. The practices recorded from local informants were grouped into various categories concerning the sources of products or materials utilized and target pests. The classes involved home-utilized products or waste materials from home and appliances, food products, living organisms such as birds, animal products or wastages, synthetic products, natural resources such as pesticide plants and miscellaneous, mixed applications, local traps, and storage structures. The frequency of citation was ranged from 6 to 135 (Table 1). Our study revealed that the relative frequencies of citation ranged from 0.03 to 0.675. Further, the ten most-cited practices values ranged from 0.38 to 0.675. The highest cited practice was seed drying before storage against stored product insect (135 times cited and RCF was 0.675) followed by plant parts like dried chilli or calotropis leaf or tobacco leaf or curry leaf in alone or combined application with stored commodities for long term storage (119 times cited and RCF was 0.595) (Fig. 3).

### IPPP used field and covariates

The IPPP used field is categorized into 7 categories, i.e., agriculture, horticulture, storage, agriculture + horticulture, horticulture + storage, agriculture + storage, and agriculture + horticulture + storage. Based on the response to a particular used field by explanatory variables, the data were synthesized and analyzed. Our findings revealed that the different responses toward the field of IPPP used among age classes were statistically significant (Kruskal–Wallis, χ^2^ = 83.378, *df* = 6, *p* < 0.05). Similarly, the difference was statistically significant among the different educational groups (Kruskal–Wallis, χ^2^ = 65.640, *df* = 6, *p* < 0.05). Likewise, the differences within various groups among occupation category and house type toward IPPP used field were statistically significant, i.e., (Pearson Chi-square, χ^2^ = 32.708, *df* = 6, *p* < 0.05) and (Pearson Chi-square, χ^2^ = 22.839, *df* = 6, *p* < 0.05), respectively. In contract to this, gender (Pearson Chi-square, χ^2^ = 6.877, *df* = 6, *p* = 0.332) and location of the informants (Pearson Chi-square, χ^2^ = 7.983, *df* = 6, *p* = 0.239) were not significant statistically for IPPP used field. Further, the findings succinct that highest used field in IPPP was noticed in agriculture + horticulture + storage for any groups within any specific explanatory variables.

### Adoption of IPPP practices and covariates

The adoption of IPPP by individual informants ranged from 0 to 12. The presumed demographic factors affecting the effect in respondents as a number of IPPP adoptions were revealed through poison count regression analysis. Explanatory variables have significant effect on IPPP adoption (Omnibus test; χ^2^ = 46.005, *df* = 6, *p* < 0.05). The people living in the hilly area have adopted more number of IPPP compared to plain areas, which was statistically significant (Wald Chi-square; χ^2^ = 15.373, *df* = 1, *p* < 0.05). In the occupational category, informants engaged in agriculture adopted comparatively more IPPP than others, and the difference was statistically significant (Wald Chi-square; χ^2^ = 10.850, *df* = 1, *p* < 0.05). Likewise, the psycho-personal variable age was having significant effect on IPPP adoption (Wald Chi-square; χ^2^ = 4.329, *df* = 1, *p* < 0.05). The beta coefficient of the age factor was 0.009, positively correlated with the number of IPPP adoption. Further, the beta coefficient of location factor for hill area was 0.80 and agricultural occupation factor was 0.345, positively correlated with the number of IPPP adoption. Remaining explanatory variables such as house type (Wald Chi-square; χ^2^ = 0.053, *df* = 1, *p* > 0.05), education (Wald Chi-square; χ^2^ = 2.219, *df* = 1, *p* > 0.05), and gender (Wald Chi-square; χ^2^ = 0.524, *df* = 1, *p* > 0.05) were statistically not significant.

## Discussion

### Status and informants knowledge of IPPP used

Indigenous practices specific to plant protection have a promising role in current agriculture. They are eco-friendly, low-cost technology and can play a significant role in sustainable pest management [14]. In this study, most of the respondents in Tripura reported that the use of IPPP has increased among various covariates. Researchers such as Patel et al. (2020) and Gyawali et al. (2021) have claimed that plant protection practices based on indigenous technology should be beneficial and a critical tool for sustainable crop production with assured food safety [14, 15]. Although many improved technologies are introduced in the current world, the traditional practices kept their position promising at an effective range. The use of traditional methods for the management of insect pests serves as a better option when ecology and economy are both concerned [16]. Therefore, the use of IPPP could minimize many unwanted problems raised by indiscriminate use of synthetic pesticides and is a matter of concern.

Our findings revealed that the farmers were pretty knowledgeable about the appropriate use of available resources in pest management sustainably. The observation was found most IPPP were used in protection of storage commodity and rice crop. It is because storage is the essential part of food security and food consumption for the future [17], and rice is the major crop in the region and cultivated by the people from time immemorial and consumed as a staple food [18, 19]. Doses of IPPP were found variable among various ethnic groups and it ranged from one location to another and within the same communities. This variability in doses is mostly due to the lack of any documented information for IPPP [20]. Hence, the irrational application needed scientific evaluation and standardizing the methods at the regional and global levels. Further, they used variable doses from their own experience and based on the incidence of pests. It was observed that the management option utilizing IPPP was also variable among crops. Still, the immediate action was merely late compared to synthetic insecticides [21]. Although the results of IPPP were merely feasible concerning all the cropping system as a whole, still the action in stored commodities and certain crops was satisfactory [22]. However, the farmers reported the use of IPPP has a prominent role in insect pest management in diverse crops. Each technology has its limit and its stand having the best effects on proper placement. Though advanced pest management (ecological engineering, push–pull strategy, novel pesticides including nano formulations and entomo-pathogens, etc.) is having immediate action, it gave a range of output. Still, the IPPP was a strong tool to protect the crops against insect pests and concerning the system approach movement and environmental safety. Hence, the interaction with the informants revealed that the amalgamation of age-old practice with novel advanced technology would boost the insect pest management strategy, which will be ecologically sustainable, economically affordable, and socially acceptable [9]. The current findings revealed that the information about the IPPP technology was gained from ancestors, forefathers, seniors, or fellow farmers where it is used both in field and storage condition to protect their crops. The trick used in tackling insect pests, particularly IPPP, is a miraculous effort transcribed from ancestors since long time [23], supported our findings. The preparation of indigenous formulation and delayed results of most IPPP might be the reasons for their less popularization among farmers [24]. Constraints in desired effectiveness could be meeting by adding or synergy in combination or incorporation with suitable one was the way forward to meet the challenge and subsequently influence popularization. All the stakeholders, including plant protectionists, extension officials, and community leaders, should be aware and educate the people about the viable role of indigenous practices in pest management.

### The IPPP recorded and frequency of citation

We recorded 39 practices about indigenous plant protection strategies, and many of the technology were similar to the study based on the preliminary survey in India and across the globe. A study in Kerala recorded 116 indigenous practices in pest management [25]. Likewise, other studies from Assam have reported 58 IPPP similar to our study [26]. The current findings are also supported by Halder et al. 2018 [23], who reported 15 ITK technologies from the eastern part of Uttar Pradesh, India. Several indigenous practices were reported regarding pest management from Assam, Odisha, West Bengal, Himachal Pradesh, Uttarakhand, Haryana, Jammu and Kashmir, and many states in Indian continent [7, 28–30]. A study from Nepal has recorded many indigenous pest management strategies including some similar practices, viz. grain storage by drying, bird scarrer, and ploughing of the field [17]. In addition, another study from Nepal also corroborated our findings [15, 24]. Likewise, the study reported from the Bangladesh is also agreed with the present reports [31]. The use of traditional plant protection practices to mitigate pests is an effective method for sustainable crop production and storage shows similarities with the ethnic groups of Machakos and Bunoma countries in Kenya [32], Zambia [33], and people elsewhere in the world. Another study reported from tropical Asia reported the usefulness of indigenous practices for successful pest management in diverse crops [34] and indeed in the world. Further, our study claimed the promising role of IPPP, which remained untapped and not explored till date, particularly to Tripura territory. This could bring some novel indigenous strategies about the pest management paradigm and explore of the untouched area of the Himalayan region like Tripura.

The citation of indigenous practices might have been influenced by using methods at adjacent localities and passed from one region to another. The most cited procedures in this study also the most commonly used practices in Tripura, such as seed drying before storage to protect the grain from storage insects, keeping the stored product with various pesticide plants like leaves of calotropis, curry leaf, and/or tobacco or dried chilli as individual component or mixed application. These strategies are followed by almost every household of Tripura. The pesticide plants reported in the study have different chemical components and constituents proven in mitigating various pest issues. Drying of stored commodities killed the hidden stages of insect life stages and reduced the moisture level that checks the pest infestation further [35]. The bioactive component of chilli is capsaicin having an insecticidal activity [36]. Likewise, calotropis has active toxic principles such as alkaloids [37], nicotine compounds in tobacco [38], and some bio-active alkaloid compounds in curry leaves [39] that effectively suppresses stored pest menace. The mode of action relies on repellant, antifeedant properties of the pesticidal plant parts [40]. Ash application causes abrasion of mouthparts, viz. mandibles, thereby repelling the insects and irritation to most the chewing pests in the vegetable garden [41]. Further, it helps in preventing oviposition by creating a physical barrier [42]. The bioactive principle of neem is broad-spectrum having insect growth disruption, oviposition suppressant, sterilant, and antifeedant action [43]. Hence, the steam decoction of neem leaf has a promising role in both sucking and chewing pest management. Application of these diverse local pesticide plants (neem, tobacco, turmeric, chilli, calotropis leaves, curry leaves) from their own experience of knowledge bank puts the challenge for the researcher to investigate more precisely and produce the products for the future generation which will be climate-resilient and ecofriendly [40]. The bioactive principles were sufficient enough to bring down the pest population level efficiently. Storage structures like granary are very useful in long-term storage of various agri products [44]. These structures, viz. granary, by using local resources like bamboo plastered with mud or cow dung slurry were the uniqueness of resource-poor farmers for a long time. The current findings were agreed with [45], who reported that grain storage structures were used to ensure food safety. Rodent trapping by placing bamboo traps having rice grain as the lure is an effective tool in vertebrate pest management [46]. Catching rodents in the rice fields or around storage structures by local traps was an example of the generosity of the invention of local people. Effective management of rice leaf folder, *Cnaphalocrocis medinalis* (Guenee) and stem borer, *Scirpophaga incertulas* (Walker) due to repellant action of *Holarrhena pubescens*, is based on the alkaloid compound, i.e., conessine alleviate major pest issues in rice crop [47].

The practices with lower frequency of citation are also useful in some way: application of soil (dried/slurry form) at the whorl of the maize crop to manage the invasive pest fall armyworm (FAW), *spodoptera frugiperda* (J.E. Smith). The mode of action relies on lack of oxygen (asphyxiation) which subsequently killed the pest. This indicates the best utilization of local resources for managing new invasive pests like FAW in Tripura. Although it is the least cited method, this has been practiced by farmer’s own experience as the pest invaded the region recently on April–May 2019 [48]. Our study revealed the farmers are the sole inventor of the technology by their own experience. The undiscovered and unexplored IPPP having scientific evidence should be interpreted for better development of technology. The antixenosis action of soap and detergent spray minimizes many sucking pests in the vegetable garden [49]. One rare practice was noticed that latex of jackfruit is used to trap the rodents [50]. Moreover, our study reported two novel strategies were adopted by the ethnic people of Tripura, i.e., use of a shaft of jackfruit for luring and killing of fruit fly species in fruit orchards and soil application to manage the invasive pest, i.e., fall armyworm is new to the scientific society.

Strategic utilization of home wastage products symbolized the innovativeness of ethnic groups. The placement of home appliances (fish net) and right application of utilized products, viz. tin boxes or hookah (smoked tobacco) water at appropriate place and time, mitigated pest problem with no or minimal cost of expenses [51]. Food products like citrus/pomelo used variably in agricultural crop cultivation manage pests distinctively [52]. Rearing duck nearby cultivated fields or engaging carnivorous birds by providing natural shelter for management of harmful pests represented the knowledge of the food chain cycle in the ecological system. The findings are in tune with the result as reported by Morrison and Lindell (2012) [53], who revealed that predatory birds play an important role as top predators in restoration systems by reducing herbivorous insects and their damage to planted trees. Home wastage products and usage of animal wastages in effective pest management prove the intimacy of farmer’s wisdom in plant protection [54, 55]. Bamboo beating or bird scarrer during the ripening stage of crop kept away grain feeder birds by threatening those [56]. Moreover, the synthetic natural products like petrol, kerosene in borer control, or tire burning in insect repulsion were the most effective technology even today’s world as long practiced by cultivators [57]. Cultural practices like deep ploughing, weed removal, crop rotation, and physical control (sun drying) were important tool for preventing pest incidence [58], as myth taught prevention is better than cure. As yellow light attracts sucking pests like whiteflies, farmers used large dried colocasia leaves or banana leaves smeared with sticky material in vegetable cropping systems for trapping those [59]. Age-old practice of rice hispa management by rope pulling was the most effective all over the state.

Though the advanced technologies in the field of pest management are developed rapidly and contentious in the changing world, the importance and effectiveness of IPPP can’t be overlooked. Further, it is practically difficult to ignore the value of IPPP in pest mitigation. Hence, the efficacy of enlisted practices should be tested scientifically in detail and validation at different regions of the globe is required in an extensive way. Moreover, the better understanding of the mitigation of pest problems should be picked out from ground level and investigation is required to develop novel products or technology. Then the promising one can be integrated with novel integrated pest management strategies for sustainable agricultural production and protection.

### Field of IPPP used and covariates

The use of IPPP depends on several explanatory variables, such as age class, education level, occupation, gender, locality, and house types of the informants and methods of application that people usually followed. The sociocultural acceptance of people varies within different places and ethnic aborigines. The use of IPPP among seven categories falls in the range agriculture + horticulture + storage > horticulture + storage > agriculture + horticulture > agriculture + horticulture > storage > horticulture > agriculture. Our study supports most of the observations of Swangla et al. (2021) [60]. The relationship between IPPP used and covariates such as age, occupation, education, and house type was statistically significant among each level, whereas the gender and location of the respondents were not significant statistically. The use of indigenous plant protection practice related to pesticide plant also significant and dependent upon specific explanatory variables such as age, education, gender, etc. (Kamanula et al. 2010) [61], which support our findings.

Since the inception of agriculture, IPPP has its importance besides the other methods of pest management. The traditional knowledge and practices developed by various ethnic growers of the region as a part of their socio-economic culture have made a strong roadmap in insect pest management practiced in Tripura. It is also important that IPPP contributes to sustainable agricultural pest management through ethnic groups’ ingenuity [14]. Hence, it is the high time for the young generations to sustain and pass the strategies to the upcoming generation. The age-old practices can be cherished in the long run in mitigating the pest battle with human civilization.

### Adoption of IPPP and respondents’ characteristics

The adoption of IPPP is an important step for any region. However, the current study revealed that adoption levels ranged from 0 to 12 for any respondents. The survey from Assam revealed the adoption of ITK regards to pest management in the crop like rice ranging from 22.50% to 79.38% [62]. A similar study by Deka et al. 2017 [26] revealed the adoption of different IPPP ranged from 8.7 to 72.5%. Likewise, the findings of Devanand, and Sabapathi, 2010 [10] also supported the current study. The current study claimed predictive variables are significant concerning to the IPPP adoption. The findings from Nepal by Naharki and Jaishi, 2020 [17] partially support our result that the adoption level is significant among various groups of farmers in pest management options. High adoption of IPPP by the people of hilly areas might be indicating the utilization of available natural resources as the land is inaccessible [4]. As this land harbored many ethnic tribes, the inherited knowledge might have encouraged the ethnic growers to take up the indigenous plant protection practices [23]. In the occupation category, those practiced and followed agriculture adopted more IPPP than other people because they were aware of the practices and very interested in utilizing low cost technologies [14]. In addition, as most of the farmers under agriculture groups are resource-poor and marginal farmers, they highly depended upon ease of availability and utilization of waste products in pest management strategies [9, 63]. The older people perceived more about the adoption of IPPP symbolized the strong belief toward age-old practices [23]. There is an emergent need to disseminate a high level of public awareness and publicity for large-scale adoption of indigenous pest management strategies.

This study has proved some basic information about the field of IPPP used and adoption level for the management of the insect pests of Tripura that could aid the development and encouraging sustainable pest management measures in agriculture. These aboriginal methods must be documented and endorsed to ease the connection between the farming and the scientific community on focused insect pest issues. The farmers attempted several control measures, the majority of which they claimed were effective. However, knowledge and skills are most important in any aspect, particularly pest management. Expertise is required to make farmers aware of appropriate pest control methods and promote the legacy of such information from generation to generation.

## Conclusion

In the present era, the system demands obtaining quality food products to ensure biosafety and restoring the natural environment. But the current mindset of achieving immediate goals leads to the indiscriminate usage of pesticide resulting in environmental pollution, biomagnifications, and ecosystem disturbance pushing humankind toward a great risk of thriving. At this point, IPPP finds their way to step forward. Though the practice was age-old, there is no negative impact on the environment and completely safer for mankind. The study found that the status of IPPP is increased during the present time. The respondents were perceived pretty knowledgeable about IPPP and claimed those were perpetuated from ancestors and fellow farmers. A total of 39 indigenous practices for pest management were investigated and reported. The field of IPPP used was associated with the social and demographic variables. Likewise, the adoption of IPPP also varied with the demographic social factors of the informants. Seed drying before storage against stored product insect was the most cited practice among indigenous methods of plant protection.

Furthermore, a higher response was perceived for agriculture + horticulture + storage (IPPP used field) by ethnic groups. Out of recorded IPPP, two measures, i.e., installation of jackfruit shaft for fruit fly management and application of soil to mitigate invasive FAW issues, are reported for the first time in the present study. Utilization of various plant products, proper management of land, water, and soil, and timely incorporation of natural available resource keep the insect pests away from our agricultural ecosystem and storage condition. Since the indigenous pest management practices are golden baskets for sustainable crop protection, these may be promoted to strengthen the ongoing pest management programs. This study recommends undertaking indigenous plant protection practices adoption and used field in Tripura. The findings enumerate the significance of traditional knowledge on pest management. Believing and adopting this will boost farmers’ self-reliance and empowerment as determinants of their course toward an improved livelihood and sustainable crop cultivation. Further, concerted efforts should be made to collect and document various IPPP from unexplored regions of the globe before they become extinct. It is recommended that efforts be given to improve the knowledge, adoption, and promotion of IPPP among various stakeholders through policy interventions and engaging scientific personnel, extensions officials, and farmers.

## Data Availability

All data generated or analyzed during this survey are included in this article.

## References

[1] Chagnon M, Kreutzweiser D, Mitchell EAD, Morrissey CA, Noome DA, Van DSJP. Risks of large-scale use of systemic insecticides to ecosystem functioning and services. Environ Sci Pollut Res. 2014;22:119–34. 10.1007/s11356-014-3277-x PMC4284381 25035052

[2] Chhetry GKN, Belbahri L (2009). Indigenous pest and disease management practices in traditional farming systems in north east India, A review. J Plant Breed Crop Sci.

[3] Rao RR (1994). Biodiversity in India: floristic aspects.

[4] Dutta SK, Chatterjee D, Sarkar D, Singh SB, Boopathi T, Kuotsu R, Vikramjeet K, Akoijam RS, Saha S, Vanlalhmangaiha Dutta SK (2016). 13 Common bean (Phaseolus vulgaris L., Fabaceae) landraces of Lushai hills in India: nutrients and antioxidants source for the farmers. Indian J Tradit Knowl..

[5] Changkija S. Biodiversity of Nagaland. Kohima: Department Forest Ecology, Environment and Wildlife. 2014.

[6] FAO. Global Forest Resources Assessment. FAO Forestry Paper No. 163. Rome: Food and Agriculture Organization. 2010.

[7] Deka MK, Bhuyan M, Hazarika LK (2006). Traditional pest management practices of Assam. Indian J Tradit Knowl.

[8] Uprety Y, Asselin H, Bergeron Y, Doyon F, Boucher JF (2012). Contribution of traditional knowledge to ecological restoration: practices and applications. Ecoscience.

[9] Grzywacz D, Stevenson PC, Mushobozi WL, Belmain S, Wilson K (2014). The use of indigenous ecological resources for pest control in Africa. Food Secur.

[10] Devanand II, Sabapathi KK (2010). Adoption of indigenous plant protection practices for sustainable environment. Agric Update.

[11] Tardío J, Pardo-de-Santayana M (2008). Cultural importance indices: a comparative analysis based on the useful wild plants of Southern Cantabria (Northern Spain). Econ Bot.

[12] SPSS. IBM SPSS Statistics for Windows (Version 21.0). IBM Corp, Armonk Chicago, IL. 2015.

[13] Avcı E (2018). Using count regression models to determine the factors which affects the hospitalization number of people with schizophrenia. J Data Sci..

[14] Patel SK, Sharma A, Singh GS (2020). Traditional agricultural practices in India: an approach for environmental sustainability and food security. Energy Ecol Environ.

[15] Gyawali P, Khanal S, Joshi JR (2021). Crop protection practices in traditional agriculture in mid-hills of Western Nepal: a case of Palpa and Gulmi District. Int J Appl Sci Biotechnol.

[16] Pal S, Chakravarthy AK. Advances in pest management in commercial flowers. CRC Press. 2020.

[17] Naharki K, Jaishi M (2020). Documentation of indigenous technical knowledge and their application in pest management in Western Mid Hill of Nepal. SAARC J Agric.

[18] Halder D, Saha JK, Biswas A (2020). Accumulation of essential and non-essential trace elements in rice grain: Possible health impacts on rice consumers in West Bengal, India. Sci Total Environ.

[19] Sen S, Chakraborty R, Kalita P (2020). Rice-not just a staple food: a comprehensive review on its phytochemicals and therapeutic potential. Trends Food Sci Technol.

[20] Mushtaq A, Pathania SS, Khan ZH, Ahmad MO. Indigenous technical knowledge in pest management. 2020.

[21] Poudel S, Poudel B, Acharya B, Poudel P (2020). Pesticide use and its impacts on human health and environment. Environ Ecosyst Sci.

[22] Juneja SB (2018). Indigenous traditional knowledge (ITK) from the farmers of Gondia district regarding use of plants against stored grain pests. Int J Innov Biosci Res..

[23] Halder J, Pandey MK, Singh N, Rai AB, Singh B (2018). Perceived effectiveness of indigenous technological knowledge (ITK) of insect and vertebrate pests management in Eastern Uttar Pradesh, India. Proceedings of the Zoological Society.

[24] Khatri S, Khanal S, Kafle S (2021). Perceived attributes and adoption of Indigenous Technological Knowledge on agriculture-a case study from Bhirkot municipality of Syangja District. Nepal Cogent food agric.

[25] Husain AS, Sundaramari M (2011). Scientific rationality and evaluative perception on indigenous plant protection practices on coconut (*Cocos nucifera* L.). J Plant Crops..

[26] Deka S, Nath RK, Sehgal M, Ahuja DB, Kakoti RK, Barbora AC (2017). Indigenous Technological Knowledge (ITK) and Practices in Pest Management of Assam. Ann Plant Protect Sci.

[27] Lenka S, Satpathy A (2020). A study on indigenous technical knowledge of tribal farmers in agriculture and livestock sectors of Koraput District. Indian J Ext Educ.

[28] Pokhrel P, Laskar, N. Indigenous technical knowledge (ITK) based pest management practices for Boro paddy in Northern parts of West Bengal. 2020.

[29] Sindhu N, Malik JS (2020). Perceived effectiveness of indigenous technical knowledge (itk) in modern agriculture in Haryana State. Indian J Ext Educ.

[30] Uddin ME, Quader A, Alam MS, Pervez AK, Islam MS, Ara T (2018). Farmers’ willingness to use innovative indigenous technical knowledge for plant protection in major crop zones of Bangladesh.

[31] Emongor RA, Uside RJ. Factors affecting adoption of integrated pest management technologies by smallholder common bean farmers in Kenya: a case study of Machakos and Bungoma Counties. Asian J Agric Extens Econ Sociol. 2019;1–12.

[32] Nkunika PO (2002). Smallholder farmers' integration of indigenous technical knowledge (ITK) in maize IPM: a case study in Zambia. Int J Trop Insect Sci.

[33] Litsinger JA. A farming systems approach to insect pest management for upland and lowland rice farmers in tropical Asia. Crop Prot Strateg Subsistence Farmers. 2019;45–101.

[34] Chauhan YS, Ghaffar MA. Solar heating of seeds—a low cost method to control bruchid (*Callosobruchus spp*.) attack during storage of Pigeonpea. J Stored Prod Res. 2002;38:87–91.

[35] Nesel A, Charleston BG, Genovia JA, Paye MDE, Rosales GS. Testing the insecticidal potential of Chili pepper (*Capsicum frutescens*) fruit extract against termites (*Coptotermes gestroi*). Biotech. 2016;182.

[36] Srivastava SK, Attri BL, Hem P (2006). Indigenous wisdom for the use of Giant weed in disease and pest management. Indian J Tradit Knowl.

[37] Adul ghany S, Hena H, Ardalan S. Insecticidal effects of some aqueous plant extracts on the control of Khapra* Trogoderma granarium*, Evert. In International Conference on Chemicals, Biological, and Environmental Sciences. 201;55–70.

[38] Kostic M, Popovic Z, Brkic D, Milanovic S, Sivcev I, Stankovic S (2008). Larvicidal and antifeedant activity of some plant derived compounds to Lymantria dispar L (Lepidoptera: Limantriidae). Bioresour Technol.

[39] Pandiyan GN, Mathew N, Munusamy S (2019). Larvicidal activity of selected essential oil in synergized combinations against *Aedes aegypti*. Ecotoxicol Environ Safe.

[40] Ayyar R (1933). Some important insect problems connected with the cultivation of rice in South India. Agric Lives India.

[41] Korunic Z (1998). Diatomaceous earths, a group of natural insecticides. J Stored Prod Res.

[42] Brotodjojo RR, Arbiwati D. Effect of application of granular organic fertilizer enriched with boiler ash and neem leaves powder on plant resistance against insect pests. Int J Biosci Biochem Bioinformatics. 2016;6:152. 10.17706/ijbbb.2016.6.4.152-157.

[43] Manandhar A, Milindi P, Shah A (2018). An overview of the post-harvest grain storage practices of smallholder farmers in developing countries. Agriculture.

[44] Tiwari BK, Gowen A, McKenna B. Pulse foods processing, quality and nutraceutical applications. 2012;172–192.

[45] Thakur NS, Firake DM, Kumar D. Indigenous Traps for the management of Rodent outbreak in North Eastern Hill region of India. 2013.

[46] Xie HH, Su J, Ge XL, Dong TT, Li X, Wen HM, Sun BH (2018). Compounds with inhibitory activity on peristalsis from the seeds of *Holarrhena antidysenterica*. Nat Prod Res.

[47] Singh S, Kandpal B, Das A, Yadav GS, Devi AG, Sharma D. Fall armyworm (FAW) management in maize, Sensitization workshop on FAW management in Tripura, leaflet (ICAR RC NEH, Tripura Centre) 2019.

[48] Imai T, Tsuchiya S, Morita K, Fujimori T (1994). Surface tension-dependant surfactant toxicity on the green peach aphid, *Myzus persicae* (Sulzer) (Hemiptera). Appl Entomol Zool.

[49] Frisch S.* U.S. Patent No. 7,669,363*. Washington, DC: U.S. Patent and Trademark Office; 2010.

[50] Pavela R (2016). History, presence and perspective of using plant extracts as commercial botanical insecticides and farm products for protection against insects-a review. Plant Prot Sci.

[51] Pathak KA, Thakur NSA, Rao KR, Shylesha AN, Insect pests of crops and their management, In: Verma ND, Bhatt BP, Steps towards modernisation of Agriculture in NEH Region, ICAR, New Delhi. 2001;121–159.

[52] Morrison EB, Lindell CA (2012). Birds and bats reduce insect biomass and leaf damage in tropical forest restoration sites. Ecol Appl.

[53] Sushmita S, Alka M, Arti P (2014). Cow dung-A boon for antimicrobial activity. Life Sci Leaflets.

[54] Singh LN, Prasad A. Upland rice in Manipur. 2011;229.

[55] Dada OA, Thomas AS, Oworu OO (2012). Response of Upland Rice (*Oryza sativa* L) cultivars to split application of compost on highly weathered soil of derived savannah agro-ecology. Ann West Univ Timisoara Ser Biol.

[56] Sharma PL, Attri BS (1969). Control of apple stem borer, *Aeolesthes holosericea Fabricius* (Cerambycidae: Coleoptera). Indian J Entomol.

[57] Lale NES, Vidal S. Mortality of different development stages of* Callosobruchus maculates* F. and* Callosobruchus subinnotatus* Pic. (Coleoptera: Bruchidae) in bambara groundnut* Vigna subterranean* (L.) Verdcourt seeds exposed to simulated solar heat. Zeitschrift fur Pflanzenkrankheiten und Pflanzenschut. 2000;107(5):553–9.

[58] Elango K, Sridharan S, Saravanan PA, Balakrishnan S (2017). Relative performance of different colour laden sticky traps on the attraction of sucking pests in pomegranate. Int J Curr Microbiol App Sci.

[59] Swangla S, Vellaichamy S, Singh P, Burman RR, Priya S, Palanisamy V, Singh T (2021). A note on indigenous technical knowledge in Kinnaur and Lahaul-Spiti districts of Himachal Pradesh. Indian J Tradit Knowl.

[60] Kamanula J, Sileshi GW, Belmain SR, Sola P, Mvumi BM, Nyirenda GK, Stevenson PC (2010). Farmers' insect pest management practices and pesticidal plant use in the protection of stored maize and beans in Southern Africa. Int J Pest Manag.

[61] Majumder D, Deka SN, Pujari D, Das PK. Traditional Knowledge adopted by the farmers for management of rice pests in North bank plain zone of Assam. 2013.

[62] Laskar N, Pal PK, Roy G, Gazmer R. Documentation and Validation of Indigenous Technology with Regard to Crop Protection of Boro Paddy under Terai Region of West Bengal. Pratibha Joshi Indigenous Technologies in Plant Protection; 2016, p. 248 ICAR–National Research Centre for Integrated Pest Management, 137.

[63] Vidyasagar PSPV, Bhat SK (1991). Pest management in coconut gardens. J Plant Crops.

[64] Sridhara S. Behavioral processes relevant to management of vertebrate pests (mammals and birds) of agriculture. Etología: Revista de la Sociedad Española de Etología. 1993;3:325–38.

[65] Bharathi PVL, Ravishankar M (2018). Vegetable nursery and tomato seedling management guide for south and central India. WorldVeg Publ.

[66] Bhumannavar BS, Viraktamath CA (2012). Biology, ecology and management of fruit piercing moths (Lepidoptera: Noctuidae). Pest Manag Hortic Ecosyst.

[67] Elakkiya P, Sujeetha JAR (2011). Validation of Integrated Pest Management (IPM) modules against rice leaf folder complex in the coastal region of Puducherry. J Rice Res.

[68] Suh J (2014). Theory and reality of integrated rice–duck farming in Asian developing countries: a systematic review and SWOT analysis. Agric Syst.

[69] Tesfaye A, Gautam RD (2003). Traditional pest management practices and lesser exploited natural product in Ethiopia and India: appraisal and revalidation. Indian J Trad Knowl.

[70] Carpio C, Dangles O, Dupas S, Léry X, López-Ferber M, Orbe K, Zeddam JL (2013). Development of a viral biopesticide for the control of the Guatemala potato tuber moth *Tecia solanivora*. J Invertebr Pathol.

[71] Das D, Baruah M (2010). Sustainable practices for pest and disease management of horticultural crops. Ann Plant Prot Sci.

[72] Jilani G, Su HCF (1983). Laboratory studies on several plant materials as insect repellants for protection of cereal grains. J Econ Entomol.

[73] Li Y, Li Z, Ye M (2008). The chemical compositions and their bioactivities in the different parts of *Eupatorium adenophorum* Spreng. J Yunnan Agric Univ.

[74] Payne TL, Shorey HH (1968). Pulsed ultrasonic sound for control of oviposition by cabbage looper moths. J Econ Entomol.

[75] Agyeman YB, Baidoo S (2019). Farmers perceptions of the effectiveness of strategies for managing wildlife crop depredation in Ghana. Int J Biodiv Conserv.

[76] Prakash A, Bentur JS, Prasad MS, Tanwar RK, Sharma OP, Bhagat S, Sushil SN. Integrated pest management for rice, National Centre for Integrated Pest Management, LBS Building, IARI Campus, New Delhi, India. 2014;43.

[77] Isely D, Methods of Insect Control. Part I. (Burgess Publ., Minneapolis, Minnesota, U.S.A.). 1951; 134.

[78] Jepson WF. A critical review of the world literature on the Lepidopterous stalk borers of tropical graminaceous crops, CIE, London, UK. 1954.

[79] Saour G, Al-Daoude A, Ismail H (2012). Evaluation of potato tuber moth mortality following postharvest cold storage of potatoes. Crop Prot.

[80] Dua VK, Pandey AC, Dash AP (2010). Adulticidal activity of essential oil of *Lantana camara* leaves against mosquitoes. Indian J Med Res.

[81] Hossain MA, Awal MA, Alam MM, Ali MR, Howlader MTH, Zahan A (2021). Prevalence of insects in traditionally stored rice at farmhouses in Bangladesh. Austr J Eng Innov Technol.

